# Fatty acid and amino acid profiles of cheese, butter, and ghee made from buffalo milk

**DOI:** 10.5455/javar.2022.i579

**Published:** 2022-03-13

**Authors:** Abu Hena Md. Asif, Md. Abid Hasan Sarker, Gautam Kumar Deb, Md. Rezwanul Habib, Sumaiya Arefin, Md. Sadakatul Bari, Md. Zakirul Islam, Md. Harun-ur- Rashid, Mohammad Shohel Rana Siddiki, Umma Fatema Shahjadee, Sharmin Akter Lisa, Salma Ahmed, Mohammad Ashiqul Islam

**Affiliations:** 1 Department of Dairy Science, Bangladesh Agricultural University, Mymensingh, Bangladesh; 2 Animal Biotechnology Division, Bangladesh Livestock Research Institute, Savar, Bangladesh; 3 Department of Livestock Services, Ministry of Fisheries and Livestock, Dhaka, Bangladesh; 4 Institute of Food Science Technology IFST, Bangladesh Council of Scientific and Industrial Research BCSIR, Dhaka, Bangladesh; † These authors contributed equally to this work.

**Keywords:** Buffalo milk, fatty acids, amino acids, cholesterol, atherogenic index, thrombogenic index

## Abstract

**Objective:**

The objective was to assess the chemical composition, cholesterol, fatty acid (FAs), and amino acid (AAs) profiles of buffalo cheese, butter, and ghee.

**Materials and Methods:**

Buffalo milk (raw) was collected from the Bangladesh Agricultural University (BAU) Dairy Farm, BAU, Mymensingh-2202, Bangladesh. Cheese, butter, and ghee were prepared at the Dairy Chemistry and Technology Laboratory, Department of Dairy Science, BAU, Mymensingh, Bangladesh, and subjected to subsequent analyses. The gross nutritional composition and AAs profile of milk were analyzed prior to the manufacture of cheese, butter, and ghee. The gross nutritional composition of milk and dairy products was analyzed by applying an automated milk analyzer and the Association of Agricultural Chemists techniques, respectively. The cholesterol, FAs, and AAs contents of cheese, butter, and ghee were determined by the Bangladesh Council for Scientific and Industrial Research, Dhaka, Bangladesh. Furthermore, atherogenic and thrombogenic indices were also calculated using reference equations.

**Results:**

The results indicated that the buffalo milk is a good source of first-rate nutrients (dry matter: 16.50%, fat: 7.50%, protein: 3.75%). Findings indicated that the butter was significantly rich with (*p* < 0.05) total solids and fat where higher (*p* > 0.05) protein, carbohydrate, and minerals were found in cheese. The saponification, Reichert-Meissl, Polenski, and Kirschner values of buffalo ghee were found to be 225, 30, 1.2, and 25, respectively. A significant (*p* < 0.05) variation was found in the cholesterol content of buffalo cheese, butter, and ghee. Butter and ghee had 40.14 and 39.57 mg more cholesterol, respectively, than cheese. The results revealed identical FA profiles except for C24:0 among the three dairy products where the major FA compositions were C4:0, C14:0, C16:0, and C18:0 and C18:1 cis-9. The atherogenicity index and thrombogenicity index of cheese, butter, and ghee were statistically similar (*p* > 0.05). Butter was found with the most conducive anti-atherogenic and anti-thrombogenic characteristics due to lower saturated and higher polyunsaturated FAs. However, all the AAs concentrations were statistically higher (*p* < 0.05) in cheese than in butter and ghee.

**Conclusion:**

To conclude, buffalo cheese is superior to butter and ghee as regards nutrient density, but consumers can choose other foods based on their choice.

## Introduction

Milk and dairy products are nutrient-dense foods that contribute significantly to human consumption [1] and provide energy and substances crucial for the development of humans, particularly infants. Buffalo milk is considered a superior raw material for western and traditional dairy products. Dairy products produced with buffalo milk are excellent in the body and firmer in texture owing to the higher content of total solids (TS), protein, and fat. Buffalo milk contains a high concentration of linolenic and conjugated linolenic acids (CLA) [2], as well as fat, lactose, caseins, AAs, and calcium [3]. These nutrients offer excellent opportunities to process and develop different dairy products from buffalo milk [2]. Importantly, buffalo milk and milk products help to prevent metabolic syndrome, chronic diseases, type-2 diabetes, hypertension, and cardiovascular disease due to higher amounts of fatty acids (FAs) and amino acids (AAs) [4].

Cheese is a good source of nutrition and a delicious milk product prepared from the coagulation of casein (with entrapped fat and water) by proteolytic enzyme action. It is expedient, versatile, and offers various flavors, textures, and forms. Cheese can be classified into many groups based on appearance, preparation, ripening, and chemical composition [5]. Cheese is manufactured from different types of milk, and its quality relies on milk composition, microbial quality, preparation, and consistency [6]. Some FAs are found in ruminant cheeses like vaccenic (C18:1*t*11) and conjugated linoleic acids (CLA; C18:2 *c*9, *t*11) that are beneficial to human health [7].

Buffalo milk is rich in fat (an average of 8.3%), implying the feasibility of using buffalo milk for manufacturing fat-based dairy products. Butter is a fat-rich dairy product where the liquid phase is dissolved in the oily phase, forming a water-in-oil emulsion. It signifies the concentration of fat as the only one of the three main constituents, like protein, fat, and carbohydrates, of milk. Protein and carbohydrates are present in minor proportions, brought into butter by buttermilk residues after churning [8].

Ghee is an anhydrous dairy product containing no less than 99.5% milk-fat and is treated as the most popular fat-rich dairy product in the Indian subcontinent. Ghee is prepared from cream or churned butter from fresh or ripened cream by heating, where ripening is carried through the fermentation of milk with bacteria inherent to milk or preferred starter cultures. Ghee is composed of mixed glycerides, FAs, phospholipids, sterols, fat-soluble vitamins, hydrocarbons, small amounts of charred casein, and trace amounts of calcium, phosphorus, iron, etc. [8]. According to Ullah et al. [9], 62% of the monounsaturated fats in ghee are rich in CLA. However, buffalo milk fat is proportionally rich in rumenic acid (the principal CLA). The CLA isomers of buffalo milk are considered anti-carcinogenic, anti-atherogenic, antiobesity, and anti-diabetic components [2].

The buffalo is vastly important in Southeast Asia, and consumers are gladly consuming buffalo milk products all over the Indian sub-continent. The type of milk is majorly responsible for the sound characteristics of cheese, butter, and ghee with proximate quality, FAs, and AAs. Nutritional composition focuses on consumer preference, and country-specific data on the nutritional composition of milk or products need to be set out [10]. 

Several articles have been reported on cheese based on biochemical, microbial, and sensory evaluations prepared from the milk of different species [11,12], emphasizing nutritional sources through feeding [13], different types of coagulant [14,15], and types of culture [16]. But no work has been located with detailed FA and AA profiles. Although there is a wealth of research on the physical and processing properties, texture, nutritional value, and FAs composition of butter from cow milk [17,18], the domain is deficient in the case of butter from buffalo milk, with a focus on FAs and AAs profiles. Most of the research work has been focused on the physicochemical and nutritional aspects of ghee from different species [19], the development of the process of arjuna herbal ghee [20], and the effect of cholesterol removal on the composition of cow ghee [21]. However, not much information is available on the detailed composition of ghee with FAs and AAs profiles, whereas, consumers are becoming more apprehensive about food compositions emphasizing FAs and AAs, which may greatly affect the maintenance of human health.

Most importantly, there is insufficient research data on the chemical characteristics highlighting FAs and AAs of cheese, butter, and ghee from indigenous buffalo in the Indian subcontinent. Therefore, the objective of this study was to assess the chemical composition, cholesterol, FAs, and AAs profiles of buffalo cheese, butter, and ghee prepared from buffalo milk. This study will help people decide what to eat based on these products’ composition and nutritional differences and how they are made.

## Materials and Methods

### Ethical approval 

Animal management procedures, methods of milking, and collection of milk samples were approved by the Animal Welfare and Experimentation Ethics Committee, Bangladesh Agricultural University (BAU), Mymensingh-2202, Bangladesh [AWEEC/BAU/2020 (21)].

### Collection and analysis of raw milk 

Morning buffalo milk (*n* = 3, non-descriptive) was collected and pooled separately from the BAU Dairy Farm (24°43'46.5"N, 90°25'22.8"E), Mymensingh-2202, Bangladesh. After that, 5 l of milk was taken from mixed samples and sent to the laboratory for product manufacturing and following analyses. Buffaloes were fed individually on German grass (*Echinochloa crus-galli*) in a cut and carry system supplemented with a concentrated mixture [22]. Buffaloes had free access to clean, fresh drinking water for 24 h. Gross milk compositions were analyzed through an automated milk analyzer (Lactoscan, SLP, MILKOTONIC Ltd., Nova Zagora-8900, Bulgaria) at the Dairy Chemistry and Technology Laboratory, Department of Dairy Science, BAU, Mymensingh-2202. The cholesterol content, FAs, and AAs were assayed at the Bangladesh Council for Scientific and Industrial Research, Dhaka, Bangladesh. Table 1 shows raw buffalo milk’s chemical composition, cholesterol level, and AA composition. The FA composition of buffalo milk was previously defined by Islam et al. [4].

### Manufacturing of cheese, butter, and ghee

The products were manufactured at the Dairy Chemistry and Technology Laboratory, Department of Dairy Science, BAU, Mymensingh-2202. The brief details are presented below.

### Cheese

**Figure 1.**
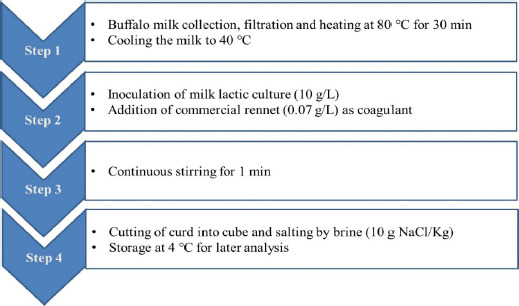
Manufacturing process of fresh cheese from buffalo milk.

The cheese-making procedure is illustrated in Figure 1. Fresh buffalo milk was heated to 80°C for 30 min before being slowly cooled to 40°C. For acidification, lactic starter culture (10 gm/l) was added by direct inoculation to milk for desirable acidity. After that, 0.07 gm/l of commercial rennet (CHY-max^®^, Denmark) was added to make the milk curd. Stirring continued for 1 min to ensure uniform incorporation of the added enzyme into the milk, followed by discontinuation to allow the curd formation. When the curd formed a clean break, it was cut into cubes and salted in brine (10 gm of NaCl/kg). The curds were placed in a perforated container and stored at 4°C for 24 h.

**Table 1.** Proximate composition, cholesterol content, and AA profile (mean ± standard deviation) of buffalo milk.

Parameters	Buffalo milk (*n* = 3)
pH	6.75 ± 0.15
Acidity (%)	0.13 ± 0.01
TS (%)	16.50 ± 0.30
Fat (%)	7.50 ± 0.26
Carbohydrates (%)	4.70 ± 0.12
Protein (%)	3.57 ± 0.11
Ash (%)	0.72 ± 0.01
Cholesterol (mg/100 g)	21.93 ± 9.36
AAs (gm 100 gm^−1^)	
Aspartic acid	0.31 ± 0.01
Threonine	0.18 ± 0.00
Serine	0.22 ± 0.01
Glutamic acid	0.78 ± 0.02
Glycine	0.14 ± 0.00
Alanine	0.19 ± 0.01
Valine	0.37 ± 0.01
Methionine	0.17 ± 0.01
Isoleucine	0.25 ± 0.01
Leucine	0.29 ± 0.01
Tyrosine	0.21 ± 0.00
Histidine	0.12 ± 0.00
Lysine	0.32 ± 0.01
Arginine	0.110 ± 0.005

**Figure 2.**
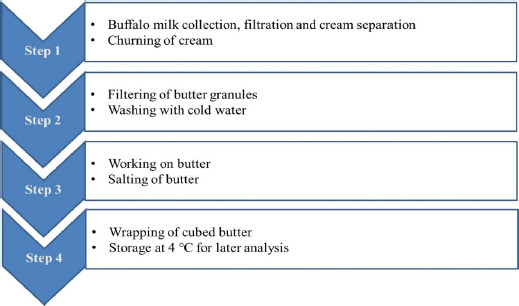
Manufacturing process of butter from buffalo milk.

### Butter

The manufacturing process of butter from buffalo milk is depicted in Figure 2. First, the cream was separated from raw buffalo milk by applying a cream separator, and its fat content was standardized to 35% (w/w). The cream was kept overnight at 4°C and churned using a 1-l capacity laboratory churn. The butter kernels were washed with cold water (12°C) in the churn. After draining butter whey, the working of butter was done to a continuous fat phase with a finely dispersed water phase to attain a homogenous mixture of butter granules, water, and salt. Approximately, 100 gm of salt (w/w) was added during the work to improve the shelf-life and flavor. The finished butter was then wrapped in cube-sized plastic and stored at 4°C for further analysis.

### Ghee

**Figure 3.**
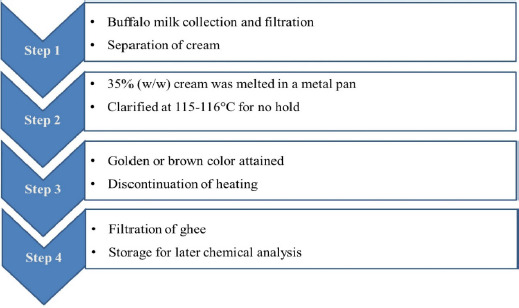
Manufacturing process of ghee from buffalo milk cream.

Manufacturing protocols for ghee from buffalo milk are shown in Figure 3. Ghee was made using the direct cream method, in which 35% (w/w) cream was melted in a metal pan and clarified at 115°C–116°C with continuous stirring until almost all of the moisture was removed. The heating was discontinued when the curd particles attained the desired golden yellow or brown color. After heating, the contents were sedimented, decanted, filtered, and packed for analysis.

### Proximate component analysis

The acidity of the cheese, butter, and ghee was measured using the titration method against 0.1 M NaOH solution and phenolphthalein indicator, and pH was measured by a pH meter-215 (Corning Diagnostic Ltd., Sudhury, Suffolk, England). The TS content was determined by evaporating at 105°C for 24 h in an oven [J.P. Selecta; S.A. ctra. Nil km: 585.1, Abrera (Barcelona) Spain]. After drying, the samples were ignited at 600°C for 6 h in a muffle furnace (Vulcan A-550, Ney^®^, USA) to estimate ash. Protein was estimated by the Kjeldahl method [23], and fat content was determined by the Babcock fat test method described by Aggarwala and Sharma [24].

### Determination of milk fat constants

#### Saponification value 

The saponification value was determined using the method Cd 3-25 [23]. First, 5 gm of filtered ghee was mixed with 50 ml of 4% alcoholic KOH, and the mixture was mildly boiled until complete saponification. After cooling, the sample was titrated against 0.5 M HCl (43.01 ml/l) in the presence of a phenolphthalein indicator (1 gm/100 ml) to neutralize excess KOH. 

#### Reichert-Meissl, Polenske, and Kirschner value

Reichert-Meissl (chiefly butyric and caproic), Polenske (mostly caprylic, capric, and lauric), and Kirschner (butyric acid) values were determined according to the method of Cd 5-40 [25]. With 5 gm of ghee sample, 20 ml of glycerol soda solution (20 ml of 500 gm/l NaOH added with 180 ml of glycerol) was added in a round-bottom flask and subsequently heated until complete saponification.

After cooling, the prepared soap solution was diluted with distilled water (135 ml) and acidified with 6 ml of sulphuric acid (H2SO4). The flask was connected to the condenser and heated to distillate volatile FAs. The collected volatile FAs were filtrated to separate soluble FAs from insoluble ones. The aqueous solution of the soluble volatile FAs and the ethanolic solution of the insoluble volatile FAs were titrated against 4 gm/l of NaOH solution in the presence of a phenolphthalein indicator (1 gm/100 ml). Then 0.5 gm of silver sulfate powder was added to the solution obtained from the RM procedure to determine the Kirschner value.

### Cholesterol analysis

#### Extraction of lipid 

In the case of milk samples, 20 ml of H2SO4 was added to 10 ml of milk and the mixture was kept in a water bath at 100°C for 30 min. To separate the fat layer, after adding 70 ml of diethyl ether, the content was shaken 4–5 times. The content was kept in a water bath at 100°C for 10 min, followed by oven drying at 102°C for 4–5 h to dry the fat layer. To keep them moisture-free, cheese, butter, and ghee samples were dried in an oven at 102°C for 4–5 h. Then further lipid extraction was done from cheese, butter, and ghee as described by the Association of Agricultural Chemists [23].

#### Analysis of cholesterol

Cholesterol analysis from extracted lipid samples of milk, cheese, butter, and ghee was done using the method Huang et al. [26] with a few modifications. A standard curve of pure cholesterol and glacial acetic acid was made (0.05 gm of pure cholesterol was dissolved into 100 ml of glacial acetic acid), and the sample measurement was done at 625 nm on a Ultraviolet-Spectrophotometer (SPECURD^®^/250 Plus, Analytikjena Co., Germany).

### Fatty acid analysis

#### Preparation of fatty acid methyl esters (FAMEs)

The Rose-Gottlieb method was applied to extract lipids from cheese, butter, and ghee samples [23]. The FA compositions of cheese, butter, and ghee were determined from their corresponding FAMEs. Ten milliliters of each extracted lipid sample were kept in a 15 ml test tube. Then, 3 ml of 0.5 M CH3ONa solution was added to the tube and digested with continuous stirring in a heating water bath for about 15 min. Subsequently, the test tube was cooled at room temperature, and 1 ml of petroleum ether with a boiling point of 40°C–60°C was added, followed by 10 ml of de-ionized. Following gentle mixing, the tubes were allowed to settle (5–6 min) the contents until the top layer of methyl ester became clear. Then, the top layer of the FAMEs was separated with care into the capped Gas chromatography (GC) vial for analysis. Two hundred milligrams of different FAs standard with corresponding methyl esters were liquefied distinctly in 10 ml petroleum ether (boiling point 40°C–60°C) in a series of screw-capped test tubes. Aliquots of 1.0 μl FAMEs were injected, and the peaks of FAs were recorded against their respective column retention times and areas with the help of the data processor unit of GC. 

#### Chromatography

FAMEs of cheese, butter, and ghee were quantified by a GC (GC; Shimadzu GC-14B, Japan) with flame ionization detector with a fused silica capillary column (FAMEWAX, Crossbond^®^ polyethylene glycol, 15 m × 0.25 mm × 0.25 μm film thickness, Restek; PA). The temperature program was kept as: the temperature at initial was kept for 5 min at 150°C after injection then programmed to increase at 190°C (at 8°C/min), kept for 10 min. Then the temperature was raised to 200°C (at 2°C/min) and held for 10 min. Both the injection and detector temperatures were 250°C, and part of a 1.0 µl volume of the sample was injected. The splitless injection technique was used with nitrogen as a carrier gas at a constant flow rate (20 ml/min). FAMEs were identified by applying a standard FAME mix (Food Industry 37 FAME mix, 35077 Restek Co, Bellefonte, PA), and areas of the peak were calculated in the chromatogram and then expressed as percentages (gm/100 g of total FA) by the automated GC software (Class GC-10, version-2.00). The atherogenicity index (AI) and thrombogenicity index (TI) was calculated following the formula recommended by Ulbricht and Southgate [27]: 

AI = [(12:0 + 4 (14:0) + 16:0] / [(n6 + n3) PUFA + 18:1 + Σ MUFA] 

TI = (14:0 + 16:0 + 18:0) / [(0.5 × 18:1) + 0.5 (Σ MUFA) + 0.5 (n6 PUFA) + 3 (n3 PUFA) + (n3 PUFA/n6 PUFA)]. 

### Amino acids analysis

Fat from milk, cheese, butter, and ghee was extracted using n-hexane (Merck, Darmstadt, Germany). Fat-free samples were then dried in an oven at 50°C for 3–5 h. For the estimation of AAs (aspartic acid, threonine, serine, glutamic acid, glycine, alanine, valine, methionine, isoleucine, leucine, tyrosine, histidine, lysine, and arginine), a dried sample of 0.5 gm was taken as described in the Shimadzu manual [28]. This AA analyzer [high-performance liquid chromatography (HPLC)] used a column (Amino-Na) to separate a highly acidic cation exchange resin. The AA adducts have been efficiently separated by using a binary gradient elution technique and detected by using a post-column derivatization detection method and a fluorescence detector at high sensitivity and pressure (88,267.1 mmHg). The HPLC system was composed of several components such as a controller (SCL-10A), liquid chromatography (LC-10AD), pump, auto-injector (SIL-10AD), column oven (CTO-10 AD), column (Amino-Na), and a fluorescence detector. Mobile A contained 0.20 M sodium citrate (adjusted pH 3.2 with perchloric acid) and 7.0% ethanol, and mobile phase B contained 0.60 M sodium citrate and 0.20 M boric acid (adjusted to pH 10.0 with 4 M sodium hydroxide). Washing solution C was composed of 0.20 M sodium hydroxide. In the fluorescence method for AAs, o-phthalaldehyde reagent, RB (o-pthalaldehyde, ethanol, polyoxyethylene lauryl ether (Brij-35), and N-acetyl-L-cysteine) were used to react with primary amines of AAs, peptides, and proteins to enable detection and quantitation. The flow rate of both RA and RB was 20–21 ml/min. To prepare the samples for AAs analysis, 0.5 gm of the oven-dried fat-free sample was crushed by the mortar pestle and mixed with 50 ml of HCl (6 M) to make a fine paste. After that, the fine paste was moved into a 250 ml round-bottom flask via filtration, and the flask containing the filtrate was placed on the heating mantle at 110°C for 22 h for hydrolysis. Thereafter, HCl was eliminated from the filtrate by adding 3–4 times distilled water and evaporating it at 90°C–95°C in a laboratory water bath. The obtained solution was filtered in a 25 ml volumetric flask using a Whatman filter paper of 41 and columned with a stock solution of 0.1 M HCl. Furthermore, that stock solution was filtered using a 0.45 µm syringe filter (Shimadzu, Japan). Then, stock and internal standard (IS) solutions (Mixed standard, Sigma-Aldrich, St. Louis, MO) were run through the AA analyzer, which displayed the standard curve for the standard solution and the sample. Finally, by comparing both the areas of two curves against stock and sample, AA was calculated in gm/100-gm as follows: 

AA= (IS concentration × Sample peak area) ÷ IS peak area.

### Statistical analysis

The independent sample *t*-test was done to determine the statistical differences in gross nutritional quality between the buffalo cheese and butter. The one-way analysis of variance was carried out to determine any significant differences among buffalo cheese, butter, and ghee in cholesterol, FAs, and AA profile. Minitab was used as statistical software (Minitab-18, Minitab Inc.^®^, State College, PA). 

## Results and Discussion

### Gross nutritional quality of cheese and butter

In Table 2, the gross nutritional substances of cheese and butter are presented. The findings indicated that all the chemical constituents of cheese and butter were significantly (*p* < 0.05) different, except for acidity and carbohydrates. Butter was found rich in TS and fat content at 29.5% and 56%, respectively. Higher TS and fat content in butter may result from variations in manufacturing technology and the percentage of chemical compositions of milk. Butter is a fat-rich milk product where fat is the major component, forming a water and oil emulsion with little carbohydrate, protein, and minerals. Due to this compositional variation, the fat content of butter is considerably higher than that of cheese. The TS and fat content of butter discovered are consistent with the findings of Hurtaud et al. [29]. However, being a protein-based product, cheese also has a greater portion of milk fat entrapped, and the fat content of buffalo cheese found in this study agrees with another study [30]. In terms of protein and mineral content, there were 21% and 3.7% higher in cheese than in butter. The higher protein and minerals in cheese are imparted due to the cheese-making process where the rennet enzymes precipitate casein. A coagulum contains casein, whey proteins, lactose, fat, and minerals. This finding agrees with Rana et al. [25], who reported 21.87% protein and 3.19% minerals in buffalo cheese. However, both cheese and butter composition conforms to the Bangladesh Standard and Testing Institute [31]. 

**Table 2.** Gross nutritional quality of buffalo milk cheese and butter (mean ± standard deviation).

Parameters	Buffalo milk		*p*-value
	Cheese	Butter	
pH	5.90 ± 0.09	6.90 ± 0.04	<0.05
Acidity (%)	0.15 ± 0.04	0.12 ± 0.05	>0.05
TS (%)	59.28 ± 5.1	88.86 ± 2.8	<0.05
Fat (%)	29.75 ± 5.88	86.00 ± 2.2	<0.05
Carbohydrates (%)	2.60 ± 0.68	0.62 ± 0.42	>0.05
Protein (%)	22.43 ± 1.6	1.46 ± 0.17	<0.05
Minerals (%)	4.50 ± 0.2	0.78 ± 0.04	<0.05

**Table 3.** Milk fat constants of buffalo milk ghee (mean ± standard deviation).

Fat constants	Buffalo milk ghee
Saponification value	225 ± 5.66
RM value	30.00 ± 4.24
Polensky value	1.20 ± 0.14
Kirschner value	25.00 ± 2.83

### Milk fat constants of ghee

In Table 3, milk fat constants signify FAs present in different kinds of fats. These are also used to detect fat adulteration qualitatively and, in some cases, quantitatively. In this study, the saponification value was 225 in ghee, implying the molecular weight of FAs. The average saponification value varies from 225.00 to 230.00 [32]. The remarkable measure of short-chain FAs implies the remarkable measure of butyric (C4) and caproic (C6) acid. The typical RM value of ruminant milk fat generally ranges from 17–35. Here, we found RM value 30 in buffalo ghee. The result is consistent with Gandhi et al. [33], who reported a range of 31.46 to 34.98 RM value in buffalo ghee. Polenske value, which typically ranges from 1.2 to 2.4 [34], denotes milk fat’s volatile water-insoluble FAs. This study found a Polenske value in buffalo ghee of 1.2, which is in the reported range. In another study, Bhatia et al. [35] found a Polenske value of 1.77 in cow ghee, suggesting more volatile water-insoluble FAs in cow milk. In this study, the Kirschner value was 25, which is also in the normal range. 

### Cholesterol and FAs composition of cheese, butter, and ghee

Table 4 summarizes the cholesterol content and FAs of buffalo cheese, butter, and ghee. A significant (*p* < 0.05) variation was found in cholesterol content among cheese, butter, and ghee, where butter appeared with the highest value (45.38 mg), followed by ghee (44.81 mg ) as fat-rich milk products. On the other hand, cheese had significantly lower cholesterol (5.24 mg) due to manufacturing technology variations. Higher fat content, buffalo milk contains significantly lower amounts of cholesterol than cow milk [36]. Buffalo milk is more popular among health-conscious people due to its lower cholesterol content. The sum of saturated FAs was found to be statistically similar (*p* > ), butter (60%), and ghee (68%), as presented in Table 4. This higher percentage of saturated FAs in all three products might be attributed to the higher saturated FAs content in raw buffalo milk [37]. Amid all the saturated FAs, only C24:0 showed significant (*p* < 0.05) variation among the products. The major saturated FAs of cheese, butter, and ghee were found to be C4:0, C14:0, C16:0, and C18:0. The FAs C4:0 (5.28%), C12:0 (2.51%), C14:0 (12.66%), and C16:0 (38.18%) were found to be high in cheese and C18:0 (13.73%) in butter. Here, it was also notable that the lower content of C16:0 had been compensated for by increased C18:1, *cis*-9, C18:2, *cis*-9, *and* 12 and C18:3, *cis*-6, 9, and 12 in buffalo butter. It is assumed that the types of FAs, *viz*., saturated, mono-and polyunsaturated, branched, and conjugated, are thought to be either positive or negative for the consumer’s health [38]. The sum of unsaturated FAs was statistically (*p* > 0.05) alike in buffalo cheese, butter, and ghee, as presented in Table 4. Oleic acid (C18:1 *cis*-9) content was found to be the second-highest in cheese (19.65%), butter (31.36%), and ghee (24.83%). This FA is very effective against cardiovascular disease and atherosclerosis [39]. In a study, buffalo milk was reported to have a higher C18:1 *cis*-9 [4]. Polyunsaturated FAs such as C18:2, *cis*-9, 12, and C18:3, *cis*-6, 9, 12, were also exponentially higher in buffalo butter. Generally, the CLA percentage of milk products largely depends on the composition of raw milk. In a study, Islam et al. [4] reported C18:2 (0.90%) and C18:3 (0.05%) among all FAs in buffalo milk. Conjugated linoleic acid isomers are thought to be anti-carcinogenic, anti-atherogenic, and anti-obesogenic [2]. The AI describes the ratio between atherogenic and anti-atherogenic FAs. Again, the TI considers the major saturated FAs as pro-thrombogenic while the unsaturated ones are considered anti-thrombogenic FAs. The differences among the atherogenic and thrombogenic indices of cheese, butter, and ghee from buffalo milk were statistically non-significant (*p* > 0.05), suggesting a positive health effect on human health. However, the AI values of cheese, butter, and ghee were 3.72, 1.65, and 2.41, respectively, where butter was recorded with the lower value. Here, atherogenic saturated FAs 12:0, 14:0, and 16:0 were found to be lower in buffalo butter with increased C18:1, *cis*-9. In this study, the AI of butter and ghee was lower than the findings of Bobe et al. [40], who reported that the high AI value in dairy products is 2.71. It is suggested that milk products with minimum AI and TI values are less likely to be negative for humans [41]. To reduce the risk of cardiovascular disease, the American Heart Association and American College of Cardiology recommended that saturated fat intake be limited to 5% to 6% of total daily calorie intake [42]. The scientific basis for reducing dietary saturated FAs has been focused on the well-established effects of rising low-density lipoprotein cholesterol, along with a decrease in non-low-density lipoprotein cholesterol, in contributing to atherosclerosis.

**Table 4.** Cholesterol content (mg 100 gm^−1^) and FA profile (% of FAMEs) of buffalo milk cheese, butter and ghee (mean ± standard deviation).

Parameters	Buffalo milk			*p*-value
	Cheese	Butter	Ghee	
Cholesterol	5.24^c^ ± 0.29	45.38^a^ ± 0.25	44.81^ab^ ± 9.70	<0.05
*ε* Saturated FAs	73.18 ± 10.37	59.90 ± 3.38	67.40 ± 3.62	>0.05
*ε* Unsaturated FAs	25.91 ± 8.57	38.01 ± 2.34	31.12 ± 3.06	>0.05
Saturated FAs				
Butyric acid (C4:0)	5.28 ± 1.19	3.62 ± 4.01	3.38 ± 3.28	>0.05
Valeric acid (C5:0)	0.55 ± 0.15	0.17 ± 0.04	1.13 ± 0.82	>0.05
Caproic acid (C6:0)	0.30 ± 0.15	0.19 ± 0.03	0.64 ± 0.29	>0.05
Caprylic acid (C8:0)	0.89 ± 0.17	0.53 ± 0.08	1.36 ± 0.43	>0.05
Capric acid (C10:0)	0.11	nd	0.09	-
Lauric acid (C12:0)	2.51 ± 0.84	0.90 ± 0.09	1.95 ± 0.45	>0.05
Tridecanoic acid (C13:0)	0.57±0.37	0.07	0.10 ± 0.02	>0.05
Myristic acid (C14:0)	12.66 ± 2.73	8.17 ± 2.24	10.43 ± 1.17	>0.05
Pentadecanoic acid (C15:0)	1.69 ± 0.08	1.57 ± 0.14	1.41 ± 0.02	>0.05
Palmitic acid (C16:0)	38.18 ± 7.97	29.01 ± 0.20	34.42 ± 0.95	>0.05
Margaric acid (C17:0)	0.75 ± 0.06	1.29 ± 0.47	0.74 ± 0.14	>0.05
Stearic acid (C18:0)	9.59 ± 1.34	13.73 ± 2.45	11.35 ± 1.52	>0.05
Arachidic acid (C20:0)	0.10	0.30 ± 0.10	0.19 ± 0.08	>0.05
Heneicosylic acid (C21:0)	Nd	0.08	0.09	-
Behenic acid (C22:0)	nd	0.14	0.06 ± 0.07	>0.05
Lignoceric acid (C24:0)	nd	0.13 ± 0.02	0.06 ± 0.01	<0.05
Unsaturated FAs				
Tridecenoic acid (C13:1)	0.47 ± 0.14	0.26 ± 0.07	0.22 ± 0.02	>0.05
Myristoleic acid (C14:1, *cis*-9)	1.77 ± 0.39	2.05 ± 0.74	1.73 ± 0.14	>0.05
Pentadecanoic acid (C15:1, cis-10)	0.41 ± 0.13	0.34 ± 0.03	0.29 ± 0.03	>0.05
Palmitoleic acid (C16:1, *cis*-9)	2.25 ± 0.98	1.72 ± 0.92	2.58 ± 0.26	>0.05
Heptadecatrienoic acid (C17:1, *cis*-10)	0.29 ± 0.06	0.18 ± 0.15	0.27 ± 0.07	>0.05
Oleic acid (C18:1, *cis*-9)	19.65 ± 7.28	31.36 ± 2.77	24.83 ± 2.51	>0.05
Linoleic acid (C18:2, *cis*-9,12)	0.75 ± 1.00	1.48 ± 0.18	0.86 ± 0.40	>0.05
Linolenic acid (C18:3, *cis*-6,9,12)	0.21 ± 0.26	0.43 ± 0.19	0.22 ± 0.11	>0.05
Eicosenoic acid (C20:1, *cis*-11 )	0.11	0.19 ± 0.08	0.12 ± 0.08	>0.05
ε Others	1.01 ± 1.32	2.19 ± 0.35	1.53 ± 0.42	>0.05
AI	3.72 ± 0.65	1.65 ± 0.02	2.41 ± 0.1	>0.05
TI	4.71 ± 0.98	2.53 ± 0.15	3.32 ± 0.21	>0.05

FAMEs, fatty acid methyl esters; nd, not detected.

^abc^ Mean values in a row with uncommon superscript letters differ significantly.

### AA composition of cheese, butter, and ghee

The quantification of AAs has a significant role in assessing the nutritional quality of foods. The AA contents of cheese, butter, and ghee are given in Table 5. Buffalo cheese provides a reliable source of AAs, and it is significantly different (*p* < 0.05) compared with butter and ghee. This higher concentration of AAs in cheese might be due to the higher protein content in cheese (22.43%) [43]. In particular, buffalo cheese is rich in essential AAs such as valine (1.25%), lysine (1.08%), and leucine (0.93%). This large amount of branched AAs confers an enormous health benefit. Moreover, the primary non-essential AAs were glutamic (3.28%), aspartic (1.53%), and serine (0.88%). On the other hand, butter and ghee are fat-rich products and contain lower amounts of AAs. Compared to ghee, butter is composed of a significantly higher (*p* < 0.05) content of AAs. It is important that glutamic acid was noticeably higher among the total AAs pool in cheese (3.28 gm), butter (0.15 gm), and ghee (0.08 gm). Glutamic acid has numerous taste perceptions, intermediate metabolism, and neurotransmission functions. Glutamic acid is considered to be a specific forerunner of arginine and proline, along with, more importantly, γ-aminobutyric acid (g amino butyric acid) and glutathione, which are anti-hypertension [44] and anti-diabetic [45].

**Table 5.** AAs profile (gm 100 gm^−1^) of cheese, butter and ghee prepared from buffalo (mean ± standard deviation)

AAs	Buffalo milk			*p*-value
	Cheese	Butter	Ghee	
Aspartic	1.53^a^ ± 0.03	0.09^b^ ± 0.01	0.05^bc^ ± 0.001	<0.05
Threonine	0.82^a^ ± 0.03	0.05^b^ ± 0.005	0.02^bc^ ± 0.001	<0.05
Serine	0.88^a^ ± 0.06	0.05^b^ ± 0.005	0.03^bc^ ± 0.001	<0.05
Glutamic	3.28^a^ ± 0.13	0.15^b^ ± 0.01	0.08^bc^ ± 0.004	<0.05
Glycin	0.67^a^ ± 0.03	0.04^b^ ± 0.003	0.02^bc^ ± 0.001	<0.05
Alanine	0.73^a^ ± 0.04	0.04^c^ ± 0.003	0.02^bc^ ± 0.001	<0.05
Valine	1.25^a^ ± 0.04	0.08^b^ ± 0.01	0.04^bc^ ± 0.001	<0.05
Methionine	0.63^a^ ± 0.06	0.03^b^ ± 0.002	0.02^bc^ ± 0.002	<0.05
Isoleucine	0.84^a^ ± 0.03	0.05^b^ ± 0.004	0.03^bc^ ± 0.001	<0.05
Leucine	0.93^a^ ± 0.03	0.07^ b^ ± 0.004	0.06^bc^ ± 0.002	<0.05
Tyrosine	0.86^a^ ± 0.03	0.05^b^ ± 0.005	0.03^bc^ ± 0.001	<0.05
Histidine	0.59^a^ ± 0.03	0.04^b^ ± 0.003	0.02^bc^ ± 0.002	<0.05
Lysine	1.08^a^ ± 0.05	0.12^b^ ± 0.006	0.07^bc^ ± 0.005	<0.05
Arginine	0.60^a^ ± 0.06	0.07^b^ ± 0.01	0.03^bc^ ± 0.002	<0.05

## Conclusion

The manufacturing process and milk types are primarily responsible for the good qualities of cheese, butter, and ghee in their nutritional profiles. The nutritional profile focuses on consumer preference, which is why buffalo milk products’ detailed compositional properties need to be set out. The chemical composition results showed that the butter was rich in proximate composition, except for protein. They found milk-fat constants were within the normal range, and these fat constants would be extremely useful in detecting adulteration of milk fat. The results also revealed that butter represents an essential source of FAs, mainly unsaturated FAs, while cheese was rich in AAs, both essential and non-essential. According to the analysis, the nutritional values of milk products differed significantly. The least atherogenicity and thrombogenicity indices were found in butter rather than cheese and ghee. Even though cheese is a protein-based milk product and butter and ghee are fat-based milk products, consumers would benefit from choosing the appropriate one based on nutritional characteristics. These findings may perhaps assist dairy farmers, milk processors, and product manufacturers with better clarification regarding the types of dairy products.

## List of Abbreviations

L, Litre; H2SO4, Sulphuric acid; μl, Microlitter; FAMEs, Fatty acid methyl esters; GC, Gas chromatography; HPLC, High-performance liquid chromatography; IS, Internal standard; LC, Liquid chromatography.
